# Purple Urine Bag Syndrome: An Alarming Hue? A Brief Review of the Literature

**DOI:** 10.4061/2011/419213

**Published:** 2011-10-01

**Authors:** Fahad Khan, Muhammad A. Chaudhry, Noorulain Qureshi, Benjamin Cowley

**Affiliations:** Section of Nephrology, Department of Internal Medicine, University of Oklahoma Health Sciences Center, Oklahoma City, OK 73126-0901, USA

## Abstract

Purple urine bag syndrome (PUBS) is a unique disease entity characterized by an alarming purple discoloration of the urine secondary to recurrent urinary tract infections with indigo- and indirubin-producing bacteria. It is usually associated with prolonged urinary catheterization and chronic debilitated states. We hereby present a concise review of this rare phenomenon with historic perspectives, epidemiology, emphasizing on current concepts of etiology, pathogenesis, relevant clinical associations, treatment modalities, prognosis, and future directions in PUBS. In addition, we highlight an interesting occurrence of this intriguing phenomenon in a 39-year-old gentleman at our institution.

## 1. Introduction

Purple urine bag syndrome (PUBS) is a rare disease entity first reported in 1978 and is signified by an alarming purple discoloration of the urine usually seen in women and chronically debilitated patients with long term indwelling urinary catheters. Recurrent urinary tract infections with bacteria containing sulphatase and phosphatase enzymes results in the formation of pigments; indirubin (red) and indigo (blue), the mixture of which turns the urine purple. We hereby present a concise review of this unique phenomenon regarding etiology, pathogenesis, clinical associations, prognosis, and future directions in PUBS.

## 2. Epidemiology

 Historically PUBS dates back to 1812 when physicians taking care of King George III noted bluish discoloration in his urine [1] and the urinary catheter and bag. King George III also had chronic constipation which is also considered as one of the major risk factors leading to PUBS. In the literature PUBS was first reported in 1978 [2]. It is an uncommon occurrence, but prevalence of PUBS has been reported to be as high as 9.8% in institutionalized patients with long-term indwelling urinary catheter use [3].

## 3. Pathogenesis

The major risk factors implicated in etiology of PUBS are outlined in Table 1. 

**Figure 1:**
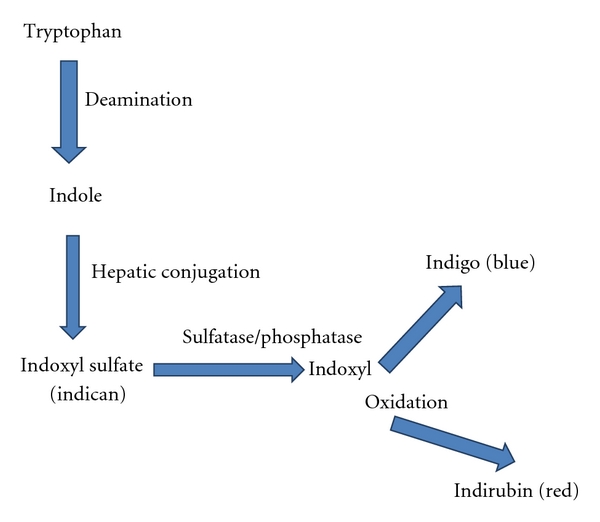
Biochemical pathway of conversion of tryptophan to indigo and indirubin.

The pathogenesis stems from multiple bacterial urinary tract infections most commonly with *Providencia stuartti and rettgeri*, *Proteus mirabilis*, *Pseudomonas auruginosa*, *Klebsiella pneumoniae*, *Escherichia coli*, *Morganella*, and* citrobacter *species, Enterococci, and Group B Streptococci. This is followed by a series of biochemical conversion reactions as shown in Figure 1 starting from deamination of tryptophan to indole, pyruvic acid and ammonia, conjugation of indole to indoxyl sulfate (indican), and oxidation of indican to indigo (blue) and indirubin (red) which combine with the catheter tubing to give purple appearance.

When oxygen is scarce, indoxyl is converted to istatin and then indirubin. In 2008, Chung reported a case of PUBS with acidic urine [6] which casts doubt on the association of urine alkalinity.

## 4. Clinical Presentations

 The patients are typically elderly nursing home women with recurring urinary tract infections. Also patients with chronic debilitation for instance in spinal cord injuries are more prone to development of PUBS. Here we would like to briefly focus on a case of PUBS at our hospital which further highlights the clinical spectrum of this phenomenon. 

**Figure 2:**
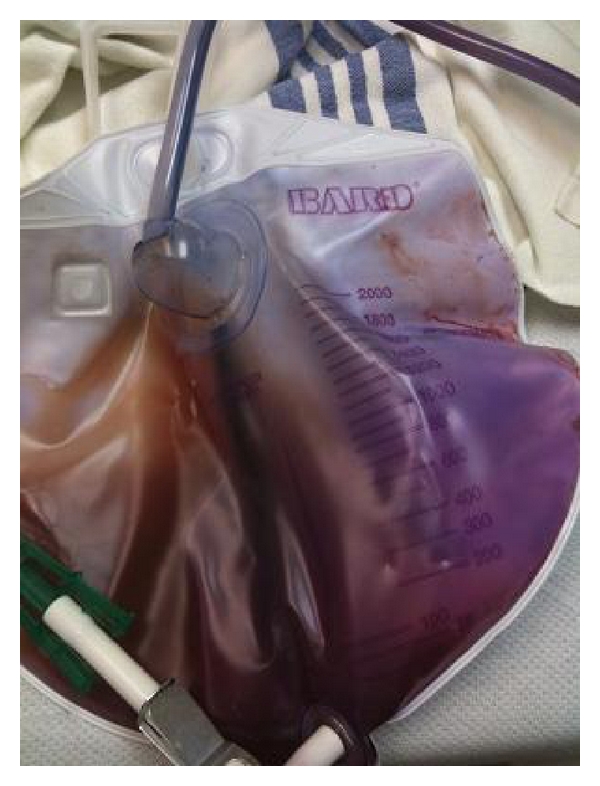
Purple discoloration of the urine with similar changes in the urine bag and tubing.

A 39-year-old gentleman was admitted to the hospital with fever, chills, and sweating since one week before admission. The patient's past medical history was significant for a motor vehicle accident in 2001 which led to injury to the cervical spine with subsequent paraplegia along with the development of a neurogenic bladder. The neurogenic bladder had been managed by indwelling suprapubic catheterization since the last eight years. The suprapubic catheter had been changed two days before admission by his wife at home. His urine culture grew four different organisms, namely, *Providencia rettgeri*, *Pseudomonas auruginosa*, *Proteus mirabilis*, and *Enterococcus faecalis*. The urine in the bag as well as the tubing was found to be purple (Figure 2). 

Upon further questioning, it was revealed that the patient had the purple urine since the last 1 year. The patient had a history of recurrent urinary tract infections (UTIs) with multiple bacteria in the past. Based on these facts he was diagnosed with PUBS and treatment for the UTI was initiated with intravenous piperacillin tazobactam and ciprofloxacin. Finally after initiating the antibiotic treatment and switching the catheter to a nonteflon one, the urinary tract infection and PUBS gradually resolved over a period of five to six days.

 There have been occurrences of PUBS in patients with chronic renal failure who are hemodialysis dependent [7] as well as in patients with nephrostomy tubes. A recent case report in 2010 showed an occurrence of PUBS in a patient with an ileal conduit and urinary diversion [8]. Another association which exists is the more frequent occurrence of PUBS with polyvinylchloride (PVC) containing urine bags as compared to non-PVC urine bags [9]. Cooccurrence with intussusception has also been described, but evidence of clear cut association is still lacking [10].

## 5. Treatment

 Treatment is directed at the underlying UTI as well as control of constipation and good urologic sanitation. Good care of the urinary catheters will prevent UTIs and hence this phenomenon as well. Though described as relatively benign, review of the literature reveals couple of cases of PUBS which progressed to Fournier's gangrene requiring aggressive debridement [11]. The typical organisms which lead to PUBS were isolated from the scrotal wound cultures. So, to treat the patient with PUBS aggressively with antibiotics is a point of debate and needs to be further investigated.

## 6. Prognosis

PUBS is generally a benign process. Despite this fact, it is distressing for family, friends, and healthcare workers who are unaware of this phenomenon and tend to become unusually alarmed because of the sudden inexplicable discoloration of the urine and sometimes the urine bag. Nevertheless, physicians should be aware of the fact that this syndrome signals underlying recurrent UTIs, due to improper care of the urinary catheters and improper sanitation. PUBS is also associated generally with higher incidence of morbidity and mortality than urinary tract infections alone without this occurrence [12].

## 7. Conclusion

 Though commonly benign, there have been case reports in the literature of PUBS progressing to Fournier's gangrene and doubts on associations of increased urine alkalinity and PVC containing urinary bags. Further definitive studies are required to elucidate clear-cut pathogenesis, morbidity, and mortality implications and to delineate standard guidelines to treat this unique phenomenon.

##  Conflict of Interests

There are no disclaimers or conflict of interests.

## Figures and Tables

**Table 1:** Risk factors and associated mechanisms in PUBS.

Risk factors for PUBS	Associated mechanisms
Female gender	Predisposing anatomy to UTI occurrence
Increased tryptophan dietary content	Increased available substrate for conversion
Increased urine alkalinity [4]	Facilitates indoxyl oxidation
Severe constipation [5]	Increased time for bacterial deamination
Chronic indwelling urinary catheterization	Increased risk of UTIs
High urinary bacterial load [5]	Bacterial sulfatase/phosphatase availability
Renal failure	Impaired clearance of indoxyl sulfate
